# Cerclage Wires Used for Extended Trochanteric Osteotomy Fixation During Two-Stage Revision Total Hip Arthroplasty for Periprosthetic Joint Infection Are Not Colonized by Bacteria: A Case Series

**DOI:** 10.1016/j.artd.2026.101978

**Published:** 2026-03-10

**Authors:** Robin Diot, Patrick Goetti, Olivier Borens, Sonia Ramos-Pascual, Julien Wegrzyn

**Affiliations:** a Department of Orthopedic Surgery and Trauma, University of Lausanne, Lausanne, Switzerland; b Bone and Motion, Clinique Bois Cerf, Hirslanden group, Lausanne, Switzerland; c ReSurg SA, Nyon, Switzerland

**Keywords:** Total hip replacement, Prosthetic joint infection, Extended trochanteric osteotomy, Cerclage hardware, Bacteria

## Abstract

**Background:**

Eradication of infection after total hip arthroplasty (THA) is particularly challenging. The aim was to evaluate whether cerclage wires, used to stabilize an extended trochanteric osteotomy (ETO) in patients undergoing two-stage revision THA for infection, become colonized with bacteria. The findings could help discern whether cerclage wires should be systematically exchanged during the second surgery to reduce the risk of persistent infection.

**Methods:**

Patients were included in the study if they had undergone two-stage revision THA for infection, that required stem removal using an ETO, and the cerclage wires had been exchanged during the second-stage procedure. The cohort comprised a consecutive series of 28 patients, 14 females and 14 males, aged 67.9 ± 13.1 years with a body mass index of 29.8 ± 6.6 kg/m^2^. Following the second-stage procedure, sonication of the explanted spacer and cerclage wires was performed separately to check for bacteria.

**Results:**

Twenty-six of the 28 patients were available for bacterial testing following the second-stage procedure. Sonication of the cerclage hardware revealed one case of bacterial contamination and no cases of bacterial colonization. Furthermore, sonication of the spacer revealed one case of bacterial contamination and no cases of bacterial colonization.

**Conclusions:**

Cerclage wires used for fixation of ETO during two-stage revision THA for infection are not colonized by bacteria, suggesting that it may not be necessary to systematically exchange them during the second-stage procedure. Further studies with larger cohort sizes are necessary to confirm these findings.

## Introduction

Periprosthetic joint infection (PJI) remains a major concern following total hip arthroplasty (THA), with an incidence of approximately 1-3% [1]. The eradication of infection after THA is particularly challenging due to the formation of bacterial biofilms on implant surfaces [2]. Thus, the gold standard for managing PJI is a two-stage revision procedure [2,3], which involves implant removal, extensive soft tissue debridement, placement of an antibiotic spacer, and a course of intravenous antibiotics, followed by implantation of a new prosthesis.

Removal of well-fixed femoral components, however, can be difficult and is associated with higher risks of intraoperative fractures, bone loss, and instability [4,5]. The use of an extended trochanteric osteotomy (ETO) has been shown to provide excellent exposure and facilitate thorough debridement [6,7], contributing to higher infection eradication with minimal increase in rates of complications [8,9]. Cerclage hardware used during the first-stage procedure to stabilize the osteotomy is often exchanged during the second-stage procedure because it can become colonized with bacteria, posing a risk for persistent infection, despite thorough debridement and antimicrobial therapy [10,11]. Nonetheless, the exchange of potentially colonized cerclage can increase surgical time, blood loss, and the risk of complications [2].

It is therefore important to investigate whether, in patients undergoing two-stage revision THA for infection, cerclage hardware used to stabilize an ETO should be systematically exchanged during the second surgery to reduce the risk of persistent PJI. The purpose of the present study was to test the cerclage wires used to stabilize an ETO to evaluate whether they become colonized with bacteria. The hypothesis was that the cerclage wires would not be colonized.

## Material and methods

A retrospective review of an institutional database was performed to identify all patients that had confirmed PJI following primary or revision THA, for surgeries performed between April 2017 and January 2021 (consecutive series). Patients were included in the study if (i) the PJI was treated using a two-stage procedure, (ii) stem removal required an ETO, and (iii) the cerclage wires were exchanged during the second-stage procedure. The cohort comprised a consecutive series of 28 patients, 14 females and 14 males, aged 67.9 ± 13.1 years with a body mass index of 29.8 ± 6.6 kg/m^2^ (Table 1). This study was approved by the institutional review board of CHUV Lausanne (number CER-VD 2016-00604).

**Table 1:** Patient characteristics, bacterial infection, and surgical data.

Variables	Mean ± SD n (%)
Women	14 (50.0%)
Age	67.9 ± 13.1
BMI	29.8 ± 6.5
ASA	2.8 ± 0.6
1	0 (0.0%)
2	9 (32.1%)
3	9 (32.1%)
4	2 (7.1%)
Diabetes	4 (14.3%)
N of prior hip surgeries	2.9 ± 1.5
Prior infection	11 (39.3%)
Bacterial culture results from first-stage procedure	
Undetermined	3 (10.7%)
Coagulase-negative staphylococci (CoNS)	1 (3.6%)
*Staphylococcus epidermidis*	7 (25.0%)
*Streptococcus anginosus*	1 (3.6%)
*Enterobacter cloacae*	3 (10.7%)
*Cutibacterium acnes*	3 (10.7%)
*Campylobacter fetus*	1 (3.6%)
*Enterococcus faecalis*	1 (3.6%)
*Escherichia coli*	2 (7.1%)
*Proteus mirabilis*	1 (3.6%)
*Rhizobium radiobacter*/*Agrobacterium fabrum*	1 (3.6%)
*Staphylococcus aureus*	6 (21.4%)
*Pseudomonas aeruginosa*	1 (3.6%)
*Klebsiella oxytoca*	1 (3.6%)
*Proteus mirabilis*	1 (3.6%)
*Candida glabrata*	1 (3.6%)
Time between THA and first-stage procedure (mo)	255.2 ± 252.1
Duration of spacer (d)	52.1 ± 36.0
Antibiotic in spacer	
Tobramycin	27 (96.4%)
Vancomycin	28 (100.0%)
Gentamicin	1 (3.6%)

ASA, American Society of Anesthesiologists; BMI, body mass index; N, number; SD, standard deviation.

### Assessment of infection

The diagnosis of PJI was based on a combination of symptoms, blood tests, and joint aspiration results, as defined by the European Bone and Joint Infection Society [12]. During the first-stage procedure, a minimum of 5 biopsy samples were taken to identify the type of bacterial infection. Furthermore, sonication of the explanted implants (cup and stem) was performed to confirm the type of bacterial infection.

During the second-stage procedure, a minimum of 5 biopsy samples were taken to check for persistent infection. Furthermore, sonication of the explanted spacer and cerclage wires was performed separately to check for bacteria. The explanted implants were placed into a sterile air-tight container (Lock&Lock, Vetrag AG, Stäfa, Switzerland) and were processed within 48 hours. Ringer solution was added into the container until it covered the whole implant. The container was then vortexed for 30 seconds, subjected to sonication (BactoSonic, Bandelin GmbH, Berlin, Germany) for 1 minute at maximum power and vortexed again for 30 seconds [13]. The resulting sonication fluid was plated in aliquots of 0.1 mL onto aerobic and anaerobic sheep blood agar plates and 1 mL was inoculated in thioglycollate broth. All cultures were incubated at 35°C for 7 days and inspected daily for bacterial growth. Microorganisms on plates were evaluated (number of colony-forming unit [CFU]/mL sonication fluid). Bacterial colonization of the implant was defined as >50 CFU/mL of any organism in the sonication fluid [14]; in contrast, bacterial contamination was defined as 1-50 CFU/mL of any organism in the sonication fluid. To prevent contamination during sample handling, standardized aseptic procedures were followed. For each sample, new sterile instruments (forceps and scalpel) were used to remove the cerclage wires. The samples were handled without direct skin contact and were immediately transferred into sterile containers. All samples were sent to the laboratory before the end of surgery for immediate bacterial testing.

### Surgical technique and antibiotic treatment

**Figure 1:**
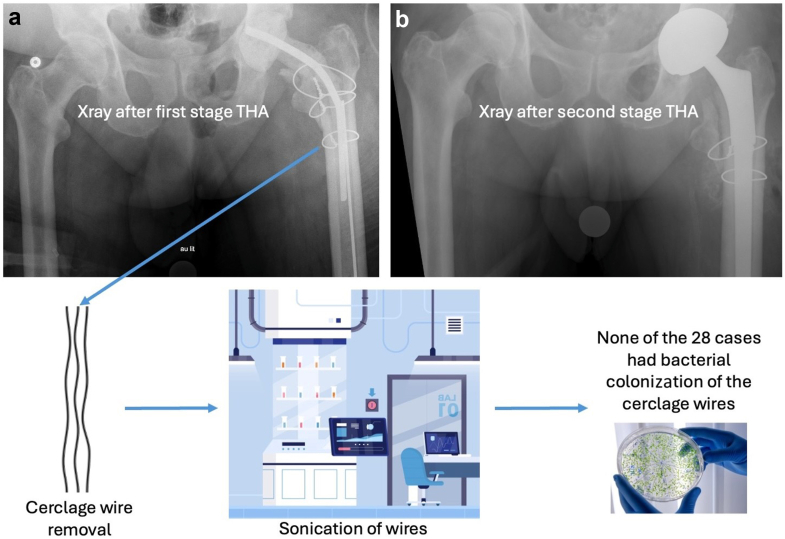
Anteroposterior radiograph of patient 21, a male aged 68 years old, (a) following the first-stage procedure showing the antibiotic spacer and cerclage wires, (b) following the second-stage procedure showing the revision total hip arthroplasty and exchanged cerclage wires.

All surgeries were performed through a posterior approach. During the first-stage procedure, extensive irrigation and debridement were performed with excision of sinus tracts (abscess or fistulas), interfacial membranes, and devitalized tissue, followed by implant removal via a Paproski ETO [6,7]. An antibiotic-impregnated cement spacer was implanted and the ETO was reduced and fixed via cerclage wires (Fig. 1). Patients were then provided with systemic targeted antibiotics based on culture results from the biopsy. The antibiotic protocol, including the length of treatment and the time between the end of antibiotic administration and the second-stage procedure, was agreed upon by the team of infectiologists and was personalized for each case based on the type of bacteria and the antibiogram. The date of the second-stage procedure was dependent on the antibiotic protocol. During the second-stage procedure, the spacer was removed, the cerclage wires were exchanged, and a new prosthesis was implanted.

### Data collection and analysis

The following were extracted from the institutional database: preoperative demographics, medical comorbidities, number of prior hip surgeries, prior infection, surgical data, as well as bacterial culture results from biopsies and sonication, and any complications, reoperations, or reinfections. Data were presented separately for each patient; in addition, descriptive statistics were performed to summarize the data.

A post hoc sample size calculation was performed to ensure adequate statistical power. Assuming a prevalence of positive cerclage cultures of 30% in 2-stage revisions [10] and a maximum infection rate of 3% in primary THA revisions [15], a one-sided Z test with 90% power and a significance level of 5% indicated that a minimum of 17 patients would be required.

## Results

The 28 patients included in the present study had undergone on average 2.9 ± 1.5 prior hip surgeries, with 11 patients having a confirmed prior infection (Table 1). The culture results from the implant sonication and biopsy samples taken during the first-stage procedure confirmed *Staphylococcus epidermidis* in 4 patients, *Enterobacter cloacae* in 3 patients, *Cutibacterium acnes* in 2 patients, *Staphylococcus aureus* in 2 patients, coagulase-negative staphylococci in 1 patient, *Streptococcus anginosus* in 1 patient, *Campylobacter fetus* in 1 patient, *Enterococcus faecalis* in 1 patient, *Escherichia coli* in 1 patient, *Proteus mirabilis* in 1 patient, *Rhizobium radiobacter* (also known as *Agrobacterium fabrum*) in 1 patient, *Pseudomonas aeruginosa* in 1 patient, *Klebsiella oxytoca* in 1 patient, *P mirabilis* in 1 patient, *Candida glabrata* in 1 patient, and undetermined bacteria in 4 patients. The time between THA and implant removal (first-stage procedure) was 255.2 ± 252.1 months, while the duration of the spacer (time between the first- and second-stage procedures) was 52.1 ± 36.0 days.

One patient died between the first- and second-stage procedures (patient 11), while one patient had no sonication results (patient 15), thus 26 of the 28 patients were available for bacterial testing at the second-stage procedure. Sonication of the cerclage hardware revealed that only 1 of the 26 patients had an infected wire (patient 1), although it was a bacterial contamination; furthermore, the bacteria in the wire (*C acnes*) was different than that originally diagnosed during the first-stage procedure (coagulase-negative staphylococci) (Table 2). Sonication of the spacer revealed that only 1 of the 26 patients had an infected spacer (patient 18), although similarly, it was a bacterial contamination; the bacteria identified was *C acnes*, which was the same bacteria identified during the first-stage procedure. There were no cases with bacterial colonization of the cerclage hardware or prosthetic implants. The biopsy samples taken during the second-stage procedure were positive in 2 patients, although both samples were considered as bacterial contamination. Patient 22 was diagnosed with *Bacillus pumilus*, but during the first-stage procedure they had *S aureus*; while patient 25 was diagnosed with *S epidermidis*, but during the first-stage procedure the type of bacteria had been undetermined.

**Table 2:** Outcomes following two-stage surgery.

Variables	Mean ± SD n (%)
Positive bacterial culture results from second-stage procedure	
Spacer (sonication)	1^a^ (3.6%)
Cerclage (sonication)	1^a^ (3.6%)
Positive biopsy	2^a^ (7.1%)
Time between second-stage procedure and last follow-up (mo)	33.4 ± 21.3
Complications	
Sciatic nerve injury	2 (7.1%)
Vascular injury	2 (7.1%)
Wound dehiscence	3 (10.7%)
Reinfection	3 (10.7%)
Rerevision for infection	2 (7.1%)

SD, standard deviation.

a Bacterial contamination.

At a follow-up of 33.4 ± 21.3 months from the second-stage procedure, a total of 10 (38%) complications were noted, including 2 (8%) sciatic nerve injuries (both transient palsies), 2 (8%) vascular injuries, 3 (12%) wound complications, and 3 (12%) recurrent or new infections (Table 2). The 2 vascular injuries were at the gluteal artery, one occurred intraoperatively and was clipped, while the other was noted immediately postoperatively and was embolized. There were no superficial or deep femoral artery injuries, nor femoral nerve injuries. Furthermore, there were no mechanical complications or cases of nonunion. The 3 wound dehiscence required debridement and resuturing, but afterward healed without issue. Regarding the 3 reinfections, 2 were caused by different types of bacteria than what had been identified during the first-stage procedure: patient 7 had been first diagnosed with *E faecalis* but was then infected with *Streptococcus mitis*; patient 14 had been first diagnosed with *S epidermidis* but was then infected with *K oxytoca* and *Staphylococcus saprophyticus*; while patient 20 had been first diagnosed with *S epidermidis* and *E cloacae* but was then infected with a combination of 5 bacteria including *E faecalis*, *P aeruginosa*, *S epidermidis*, *Staphylococcus hominis*, and *Staphylococcus haemolyticus*. Of these 3 patients with a reinfection, 1 underwent rerevision with an articulated spacer (patient 7), 1 underwent Girdlestone resection (patient 14), while the other underwent lavage and debridement (patient 20).

## Discussion

The present study on patients undergoing two-stage revision THA with ETO for infection evaluated whether the cerclage wires used to stabilize the ETO become colonized with bacteria. The most important finding was that none of the cerclage wires were truly colonized with bacteria, although bacterial contamination was detected in the cerclage wire of 1 of the 28 patients. Therefore, the hypothesis that cerclage wires would not be colonized by bacteria was confirmed. The findings of the present study suggest that cerclage wires do not become colonized with bacteria, and therefore it may not be necessary to systematically exchange them during the second-stage procedure; thus reducing surgical time, blood loss, and the risk of complications. Nonetheless, future studies with larger cohorts are necessary to confirm these findings before the current surgical protocols can be changed, and the routine exchange of cerclage wires can be omitted.

The present study, which included a consecutive series of patients, found a recurrent or new PJI rate of 11% at a follow-up of 33.4 ± 21.3 months, which is similar to that reported in the literature for studies on revision THA with cerclage fixation for PJI at similar follow-ups (4%-20%) [2,9,11]. Additionally, the cohort had comparable patient characteristics (age, sex distribution, comorbidities) to similar published articles [2,11], thus the cohort is likely representative of the broader population undergoing this procedure. Furthermore, the present study found no evidence of true bacterial colonization of the implants, although bacterial contamination was detected in one (4%) cerclage wire and one (4%) spacer. It is interesting to note that, in the case that revealed a positive sonication of the spacer, the sonication of the cerclage of that same patient was negative. This could be because the spacer was contaminated as believed by the infectiologists, but if this was not the case, it would suggest that even in cases where the spacer is infected, the cerclage may not be, possibly because it is distal to the infected joint.

Whittaker et al. [2] evaluated patients who needed two-stage revision with ETO for PJI following a THA or hip hemiarthroplasty and compared outcomes of patients who had cerclages retained (n = 40) during the second-stage vs those who had cerclages exchanged (n = 9). The authors found little to no differences in rates of new or recurrent PJIs (20% vs 22%; *P* = .881), although patients with cerclage retention tended to have higher rates of repeat revision for infection (18% vs 11%; *P* = .639), but tended to have lower rates of overall surgical complications (35% vs 56%; *P* = .253), subsidence (3% vs 11%; *P* = .238), and dislocation (10% vs 22%; *P* = .312); however, the group with cerclage retention had a significantly longer follow-up (3.3 years vs 1.0 years), thus it is possible that at comparable follow-ups the rates for revision of repeat infection would be similar. The cohort sizes were also considerably small (40 vs 9 patients), and the cerclage hardware used included wires, cables, and sutures. In fact, Whittaker et al. [2] performed a second analysis and found that ETOs fixed with cables tended to have higher rates of new or recurrent PJIs compared to ETOs fixed with wires (30% vs 18%; *P* = .422). Furthermore, other studies on revision THA for PJI using ETO have reported high infection eradication rates and low complication rates, but outcomes varied depending on whether cerclage wires were exchanged or retained, with some authors suggesting a trend toward improved infection control with cerclage exchange, though not statistically significant in all cohorts [8,9,16]. Additionally, in patients with co-existing fractures treated with spacer-less Girdlestone resection arthroplasty and cerclages, a trend of higher rates of early reinfection, relapse by the same microbe, and infection with difficult-to-treat organisms were observed, suggesting that retained cerclage hardware is a potential source of ongoing infection, although results were not significant, possible due to the small cohort size [11]. Discrepancies in outcomes across studies may be attributable to differences in patient characteristics, diagnostic criteria, and surgical technique.

Sonication has demonstrated to be reliable and sufficient for pathogen detection in clinical diagnostic routine [[17], [18], [19]]. Furthermore, a number of studies that have investigated bacterial adherence to joint prostheses have found that a biofilm was present on all intra-articular components, with 2 studies reporting the highest bacterial load on polyethylene liners, and 1 study reporting no differences in adherence to a particular component or a particular biomaterial [[17], [18], [19]]. Additionally, these studies also reported that *S epidermidis* and *S aureus* were the most common isolated organisms. In the present study, cerclages and spacers were sonicated separately to avoid elution of antibiotics, which could lead to elevated concentrations of antibiotics in the sonication fluid, inhibiting bacterial growth [20]. However, infectiologists believed that the 2 positive cases from sonication following the second-stage procedure (1 positive cerclage and 1 positive spacer) were contaminations, due to the very small concentration of bacteria. To confirm this, cerclages and spacers could have been sent for examination under an electron microscope, as they have increased sensitivity for detection of bacteria, which may not be distinguished during sonication.

The present study included a cohort of patients with PJI who were treated with a 2-stage procedure using the implantation of a cement spacer. However, instead of implanting a spacer, some surgeons perform a Girdlestone resection to debride the infected area, leaving a crude articulation between femur and acetabulum, as well as resulting in a temporary limb length discrepancy. Janz et al. [10] performed a true Girdlestone resection arthroplasty in 23 patients who underwent 2-stage THA revision for infection with either ETO or other femoral osteotomy and found that 7 patients (30%) had bacterial colonization of the cerclage hardware, with 2 of these patients (29%) requiring rerevision THA due to septic complications. In contrast, the present study found much lower rates of cerclage colonization, which could suggest that the presence of an antibiotic-eluting spacer may decrease the rate of bacterial colonization of the in situ cerclage hardware. A Girdlestone resection may have been preferred over implanting a cement spacer because older studies [[21], [22], [23]] have associated cement spacers with a higher rate of mechanical complications, such as spacer breakage or dislocation, additionally spacers could be contraindicated in patients with severe acetabular osseous defects; however, in the present study, there were no mechanical complications, albeit the small cohort size. Additionally, future trends in treatment of PJI may move toward 1-stage instead of 2-stage THA revision, which can offer shorter surgical times, less blood loss, reduced exposure to anesthetic and surgical risks, lower rates of complications, as well as better cost-effectiveness [[24], [25], [26]]. Nonetheless, it remains to be confirmed whether 1-stage procedures are as effective in decreasing the risk of reinfection.

Although the findings from the present study suggest that it may not be necessary to systematically exchange cerclage wires, some authors believe there are risks involved with the retention of cerclage wires in the setting of PJI [2,11]. The concern for persistent infection arises due to the potential for biofilm formation and bacterial colonization, even when wires are presumed to be free of bacteria at the time of the second-stage procedure. This could be particularly relevant in the context of multiresistant pathogens, which are associated with higher rates of treatment failure and early recurrence [11,[27], [28], [29]]. However, there is no clear evidence to confirm that systematically exchanging cerclage wires decreases the rates of infection; and furthermore, the routine exchange of cerclage hardware can increase surgical time, blood loss, and the risk of complications [2].

Prior infections, antibiotic exposure, and bacterial strain differences can all considerably influence bacterial colonization and detection, although only antibiotic use is a modifiable risk factor for both colonization by resistant organisms and altered pathogen detection profiles. Prior infections can alter host immunity and the local microenvironment, potentially increasing susceptibility to colonization by new or resistant organisms [30]. Furthermore, antibiotic use disrupts the native microbiota, reducing colonization resistance and facilitating overgrowth or colonization by resistant or opportunistic pathogens. Multiple studies have demonstrated that prior antibiotic exposure significantly increases the risk of colonization with multidrug-resistant bacteria, with effects persisting for months to years after exposure [[31], [32], [33]]. Furthermore, bacterial strain differences affect colonization dynamics and detection. Strain-specific factors, including resistance gene carriage and metabolic adaptability, influence both colonization success and the likelihood of subsequent infection. It is also important to note that prior antimicrobial therapy, especially if it is prolonged for more than 14 days, can reduce the sensitivity of sonication fluid cultures for detecting implant-associated infections, especially if antibiotics are administered within 14 days before sampling [[34], [35], [36]]. Combining results from sonication fluid and tissue cultures improve overall diagnostic accuracy, as each method may identify pathogens missed by the other [34,37]. In the present study, for most of the patients (26 of 28 patients), the time between the first- and second-stage procedure was >14 days, which could have decreased the incidence of detection of bacterial growth on the cerclage wires. Future studies should consider the use of other technologies, such as electron microscopy, which may improve the accuracy of bacterial detection.

The present study should be read with the following limitations in mind. First, this was a retrospective study, which inherently relies on pre-existing medical records that may be subject to incomplete or inaccurate documentation, as well as recall bias. Second, the present study had a small sample size of 28 patients, thus these results should not be generalized, and a larger cohort is needed to confirm the findings. Nevertheless, it included a niche population of patients with PJI undergoing 2-stage revision THA with ETO, which explains the small sample size. Third, all patients were operated in the same center by experienced surgeons, which may limit the generalizability of the results. Third, there was heterogeneity in terms of patient characteristics and comorbidities, bacterial infection, antibiotic protocol applied, and duration of the spacer. Nonetheless, this represents the differences that are usual in clinical practice. Fourth, the same surgical approach was used for all patients, although this makes the cohort more homogeneous, it also decreases the generalizability of the study. Fifth, there may be potential detection bias due to the use of sonication. Although the combination of both sonication of the implants and biopsy samples taking during the surgical procedures, which is recommended by the Infectious Disease Society of America and supported by recent literature [34,37], should have increased the sensitivity for pathogen detection.

## Conclusions

Cerclage wires used for fixation of ETO during 2-stage revision THA for infection are not colonized by bacteria, suggesting that it may not be necessary to systematically exchange them during the second-stage procedure. Further studies with larger cohort sizes are necessary to confirm these findings.

## Funding

Centre Hospitalier Universitaire Vaudois provided funding for data analysis and manuscript writing.

## Ethical approval

This study was approved by the institutional review board of “Commission cantonale d’ethique de la recherche sur l’être humain” of Vaud (CER-VD; BASEC number: 2016-00604).

## Conflicts of interest

J. Wegrzyn received royalties from Dedienne Santé; received consulting fees from Stryker and Enovis; and is in the Editorial board of *Swiss Medical Weekly and Journal of Arthroplasty*; all other authors declare no potential conflicts of interest.

For full disclosure statements refer to https://doi.org/10.1016/j.artd.2026.101978.

## CRediT authorship contribution statement

**Robin Diot:** Writing – original draft, Project administration, Methodology, Formal analysis, Data curation, Conceptualization. **Patrick Goetti:** Writing – review & editing, Supervision, Data curation, Conceptualization. **Olivier Borens:** Writing – review & editing, Validation, Supervision, Data curation, Conceptualization. **Sonia Ramos-Pascual:** Writing – original draft, Visualization, Software, Formal analysis. **Julien Wegrzyn:** Writing – review & editing, Resources, Data curation, Conceptualization.

## Appendix A Supplementary data

Conflict of Interest Statement for Wegrzyn

Conflict of Interest Statement for Borens

Conflict of Interest Statement for Goetti

Conflict of Interest Statement for Diot

Conflict of Interest Statement for Ramos-Pascual
